# Isoschaftoside Inhibits Lipopolysaccharide-Induced Inflammation in Microglia through Regulation of HIF-1*α*-Mediated Metabolic Reprogramming

**DOI:** 10.1155/2022/5227335

**Published:** 2022-11-23

**Authors:** Shuyuan Guan, Lingbin Sun, Xihua Wang, Xirui Huang, Tao Luo

**Affiliations:** Department of Anesthesiology, Peking University Shenzhen Hospital, Shenzhen 518036, China

## Abstract

Isoschaftoside is a C-glycosyl flavonoid extracted from the root exudates of *Desmodium uncinatum* and *Abrus cantoniensis*. Previous studies suggested that C-glycosyl flavonoid has neuroprotective effects with the property of reducing oxidative stress and inflammatory markers. Microglia are key cellular mediators of neuroinflammation in the central nervous system. The aim of this study was to investigate the effect of isoschaftoside on lipopolysaccharide-induced activation of BV-2 microglial cells. The BV-2 cells were exposed to 10 ng/ml lipopolysaccharide and isoschaftoside (0–1000 *μ*M). Isoschaftoside effectively inhibited lipopolysaccharide-induced nitric oxide production and proinflammatory cytokines including iNOS, TNF-*α*, IL-1*β,* and COX2 expression. Isoschaftoside also significantly reduced lipopolysaccharide-induced HIF-1*α*, HK2, and PFKFB3 protein expression. Induction of HIF-1*α* accumulation by CoCl_2_ was inhibited by isoschaftoside, while the HIF-1*α* specific inhibitor Kc7f2 mitigated the metabolic reprogramming and anti-inflammatory effect of isoschaftoside. Furthermore, isoschaftoside attenuated lipopolysaccharide-induced phosphorylation of ERK1/2 and mTOR. These results suggest that isoschaftoside can suppress inflammatory responses in lipopolysaccharide-activated microglia, and the mechanism was partly due to inhibition of the HIF-1*α*-mediated metabolic reprogramming pathway.

## 1. Introduction

Microglial cells are the resident macrophages in the central nervous system, and they provide the first line of defense against brain injury. They become activated in response to central nervous system injury or damage [1]. The classic activation cascade of microglia can promote the synthesis and secretion of a large number of inflammatory mediators including nitric oxide (NO), proinflammatory cytokines interleukin-1 (IL-1), IL-6, and tumor necrosis factor-*α* (TNF-*α*), leading to neuronal death and accelerating the pathological progress of neurodegenerative diseases [2, 3]. Suppressing microglial overactivation is considered to be a promising therapeutic to a wide variety of CNS diseases.

Recent studies suggest that microglia activation can be regulated by their metabolism [4]. During the classic inflammatory activation process, microglia cellular metabolism as well as energy production is reprogrammed from oxidative phosphorylation (OXPHOS) toward glycolysis even in the presence of oxygen, a phenomenon also known as the Warburg effect [5]. Inhibiting the glycolytic metabolism of activated microglia proved to be an effective therapeutic target for neuroinflammation and neurodegenerative diseases [6–8].

Isoschaftoside is a C-glycosyl flavonoid extracted from the root exudates of *Desmodium uncinatum* and *Abrus cantoniensis* [9, 10]. Previous investigations have demonstrated that C-glycosyl flavonoid has pharmacological activity for hepatic protection against D-galactosamine (D-GalN)-induced hepatitis, carbon tetrachloride (CCl_4_)-induced hepatic fibrosis, and high-fat diet-induced nonalcoholic fatty liver disease (NAFLD) in rats [9]. C-glycosyl flavonoid is also capable of attenuating AlCl_3_-induced neurotoxicity in rats through inhibition of oxidative stress and inflammatory markers [11]. However, little is known about the effect of isoschaftoside on neuroinflammation in the central nervous system. The objective of the present study was to investigate the effect of isoschaftoside on the inflammatory response induced by lipopolysaccharide and its molecular mechanism in cultured BV2 microglial cells in vitro.

## 2. Materials and Methods

### 2.1. BV2 Microglial Cell Culture

The murine BV2 microglial cells were purchased from China center for type culture collection (Wuhan, China) [11]. BV2 cells were cultivated in the DMEM medium supplemented with 10% FBS and 1% P/S in a humidified 5% CO_2_ atmosphere at 37°C.

### 2.2. Cell Treatment

BV2 cells were treated with or without LPS (10 ng/mL), in the presence or absence of isoschaftoside (200 *μ*M) [12]. For the cellular signaling pathway study, BV2 cultures were pretreated with CoCl_2_ (100 *μ*M) and Kc7f2 (10 *μ*M), respectively, followed by lipopolysaccharide (10 ng/mL) and/or isoschaftoside (200 *μ*M) treatment accordingly. Lipopolysaccharide, CoCl_2_, and DMSO were obtained from Sigma-Aldrich (Shanghai, China). Kc7f2 was purchased from SELLLECK (Shanghai, China). Isoschaftoside, an allelopathic di-C-glycosyl flavonoid from Desmodium spp. root exudates and Abrus cantoniensis, is biosynthesised through sequential glucosylation and arabinosylation of 2-hydroxynaringenin with UDP-glucose and UDP-arabinose [13] and was obtained from Med Chem Express (New Jersey, USA).

### 2.3. Cell Viability Assay

Cells were cultured in a 96-well plate. After experimental treatment, a cell count kit-8 was used to quantitatively evaluate the cell viability according to the manufacturer's instructions (Beyotime, China), and then the plates were incubated in the 5% CO_2_ incubator for 2 h at 37°C. The absorbance was determined at 450 nm using a microplate reader.

### 2.4. Measurement of Nitrite Production

The Griess reagent (Beyotime, China) was used to detect the concentration of NO in the cell culture supernatant. BV2 cells were seeded in 96-well plates and treated with 0–1000 *μ*M isoschaftoside and 10 ng/mL lipopolysaccharide for 24 h. The supernatant was collected and made to react with the Griess reagent at room temperature for 30 minutes. The absorption of the mixture was measured at 540 nm using a microplate reader.

### 2.5. Western Blot Analysis

RIPA buffer containing the protease inhibitor (Beyotime, China) was used to lyse the treated cells on ice, and the protein concentration was detected by using the BCA protein quantitative kit. Equivalent amounts of total proteins were separated by 12% SDS-PAGE and transferred to a polyvinylidene fluoride (PVDF) membrane (MILLIPORE, China). After blocking with 5% deproteinized milk powder at room temperature for 2 h, the membranes were incubated with the corresponding primary antibody at 4°C overnight. This was followed by horseradish peroxidase-conjugated secondary antibodies at room temperature for 2 h. The protein contents were determined by the chemiluminescence detection system (Tanon 5200), and the results were analyzed by the ImageJ analysis system. Antibodies including anti-iNOS (A18247), anti-HIF-1*α* (A16873), anti-HK2 (A0994), anti-COX-2 (A1253), anti-*β*-actin (A-C026), and HRP goat anti-rabbit IgG (H + L) were obtained from Abclonal (Wuhan, China). Anti-IL-1*β* (ab9277), anti-PFKFB3 (ab181861), and anti-TNF-*α* (ab6671) were obtained from Abcam (Cambridge, UK). Anti-phospho-p44/42 MAPK (Erk1/2) antibody (#9101), anti-p44/42 MAPK (Erk1/2) antibody (#9102), anti-phospho-mTOR (#5536), and anti-mTOR (#2983) were from Cell Signaling Technology (Trask Lane, USA).

### 2.6. In Vitro Immunofluorescence

BV2 cells were cultured on gelatin-coated glass coverslips until fusion. Cells were induced with cobalt chloride for 24 h and then treated with isoschaftoside for 9 h. The cells were washed twice in PBS and fixed with 4% paraformaldehyde for 10 minutes. Polyformaldehyde was removed by washing three times in PBS and then penetrated for 5 minutes with 0.2% Triton X-100 and 5% BSA in PBS for 30 minutes. The slides were incubated with the primary antibody at 4°C overnight, followed by the goat IgG (1 : 200)-Alexa fluor488 secondary antibody, and they were then restained with DAPI (1 *μ*g/ml). The bx530 Olympus microscope with Olympus dp47 digital camera was used for photo taking, and the fluorescence intensity was analyzed by ImageJ.

### 2.7. Statistical Analyses

Statistical analyses were determined by using GraphPad prism software 6 (La Jolla, CA), and the data are expressed as means ± SD. Two-way ANOVA was used for statistical comparisons followed by Tukey's post hoc test. Statistical significance was set at *P* < 0.05.

## 3. Results

### 3.1. Isoschaftoside Attenuates Lipopolysaccharide-Stimulated Proinflammatory Mediator Production in Microglial Cells

**Figure 1:**
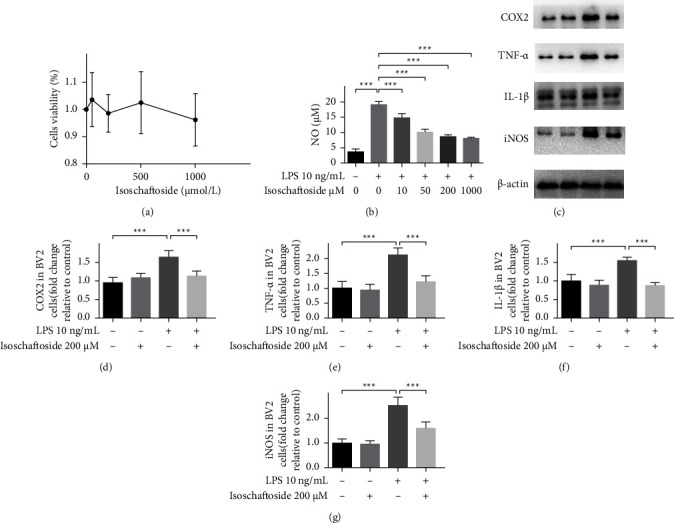
Isoschaftoside downregulates lipopolysaccharide-stimulated expression of pro-inflammatory mediators. (a) CCK-8 assay shows that isoschaftoside did not have cytotoxic activities on BV2 cells within a range of concentrations. (b) The production of nitric oxide in the culture medium was quantified by Griess assay. (c–g) The cell lysates were obtained for expression of COX2, TNF-*α*, IL-1*β* and iNOS by western blot. The data are expressed as means ± SD; ^*∗∗∗*^*P* < 0.001 for comparisons shown, *n* = 6. Statistical analyses were done by two-way ANOVA with the Tukey's post hoc test.

Isoschaftoside at concentrations up to 1000 *μ*M had no cytotoxic activities on BV2 cells (Figure 1(a)). To explore if isoschaftoside has anti-inflammatory effects, BV2 cells were treated with various concentrations of isoschaftoside (0–1000 *μ*M) and stimulated with 10 ng/mL lipopolysaccharide for 24 h. The results showed that isoschaftoside inhibited lipopolysaccharide-induced nitric oxide production in a dose-dependent manner (Figure 1(b); *P* < 0.001 versus LPS). When BV2 microglia were exposed with lipopolysaccharide, the protein levels of iNOS, TNF-*α*, and IL-1*β* and COX2 protein levels were significantly increased compared with the untreated control (*P* < 0.001 versus control, Figures 1(c)–1(g)). Isoschaftoside (200 *μ*M) significantly inhibited lipopolysaccharide-induced iNOS, TNF-*α*, IL-1*β*, and COX2 protein expression (*P* < 0.001 versus LPS, Figures 1(c)–1(g)). These data indicate that isoschaftoside plays an anti-inflammatory role in lipopolysaccharide-activated BV2 microglial cells.

### 3.2. Effect of Isoschaftoside on Lipopolysaccharide-Stimulated Expression of HIF-1*α* and Glycolytic Enzymes

**Figure 2:**
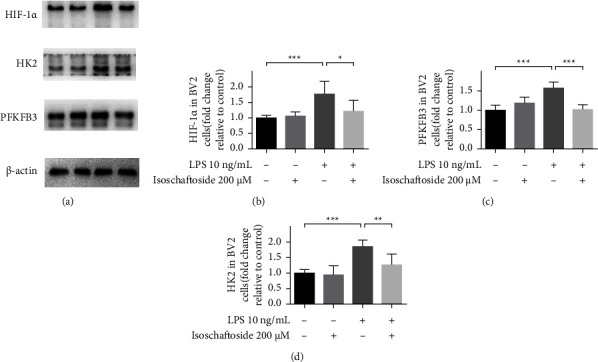
Isoschaftoside inhibits lipopolysaccharide-induced HIF-1*α* and glycolytic enzymes expression. BV2 cells were treated with lipopolysaccharide (10 ng/mL) and isoschaftoside (200 *μ*M) for 9 h (a–d) The expression of HIF-1*α*, HK2 and PFKFB3 was quantified by western blot. The data are expressed as means ± SD; ^*∗*^*P* < 0.05, ^*∗∗*^*P* < 0.01, ^*∗∗∗*^*P* < 0.001 for comparisons shown, *n* = 6. Statistical analyses were done by two-way ANOVA with the Tukey's post hoc test.

Microglia activation is featured with metabolic reprogramming based on increased glucose uptake and anaerobic glycolysis [5]. To study the underlying anti-inflammatory mechanism of isoschaftoside, we analyzed the protein expression of HIF-1*α* and glycolytic enzymes (Figures 2(a)–2(c)). Lipopolysaccharide stimulation induced upregulation of HIF-1*α*, HK2, and PFKFB3 (*P* < 0.001 versus control), while isoschaftoside significantly reduced the lipopolysaccharide-induced HIF-1*α*, HK2, and PFKFB3 expression, respectively (*P* < 0.05, *P* < 0.01, or *P* < 0.001 versus LPS).

### 3.3. HIF-1*α* Is Required for Regulating the Anti-Inflammatory Effect of Isoschaftoside

**Figure 3:**
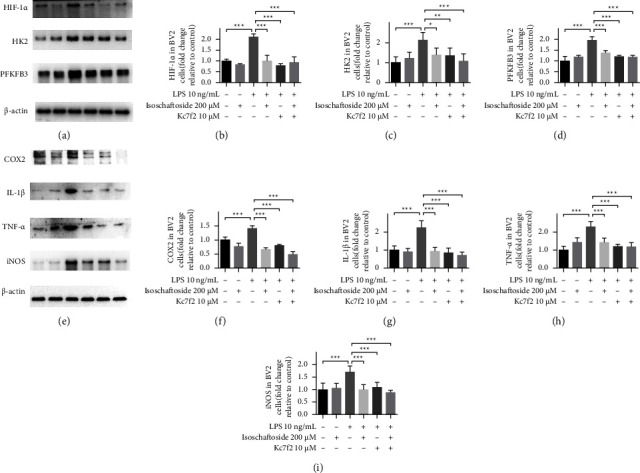
HIF-1*α* is involved in regulating the anti-inflammatory effect of isoschaftoside. BV2 cells were treatment with lipopolysaccharide (10 ng/mL) and isoschaftoside (200 *μ*M). (a–i) The expression of COX2, TNF-*α*, IL-1*β*, iNOS, HIF-1*α*, HK2 and PFKFB3 was quantified by western blot. The data are expressed as means ± SD. ^*∗*^*P* < 0.05; ^*∗∗*^*P* < 0.01; ^*∗∗∗*^*P* < 0.001 for comparisons shown, *n* = 6. Statistical analyses were done by two-way ANOVA with the Tukey's post hoc test.

**Figure 4:**
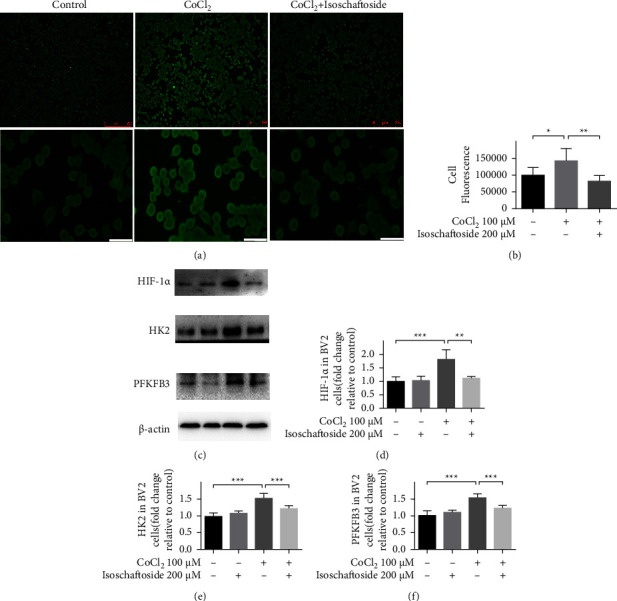
Isoschaftoside reduces HIF-1*α* and glycolytic enzymes production in CoCl_2_-treated microglia. BV2 cells were pretreated with 100 *μ*M CoCl_2_ for 24 h followed by 200 *μ*M isoschaftoside for 9 h. (a) Representative fluorescence images of HIF-1*α* was observed by immunofluorescence microscope. (Scale bar above: 250 *μ*m, scale bar below: 25 *μ*m). (b) Quantification of average fluorescence intensity of HIF-1*α* staining by ImageJ. (c–f) The expression of HIF-1*α*, HK2 and PFKFB3 by western blot. Data are expressed as means ± SD, ^*∗*^*P* < 0.05; ^*∗∗*^*P* < 0.01; ^*∗∗∗*^*P* < 0.001 for comparisons shown, *n* = 6. Statistical analyses were done by two-way ANOVA with the Tukey's post hoc test.

To investigate whether the inhibitory effect of isoschaftoside on lipopolysaccharide-induced microglia activation depends on the inhibition of HIF-1*α* expression, BV2 cells were exposed to 10 *μ*M Kc7f2 (a selective inhibitor of HIF-1*α* transcription) for 24 h, followed by incubation with lipopolysaccharide and isoschaftoside for 9 h. The results showed that in the presence of Kc7f2, the inhibitory effect of isoschaftoside on lipopolysaccharide-induced neuroinflammation did no longer exist (Figures 3(e)–3(i)). Similarily, isoschaftoside had no further effect on HIF-1*α*, HK2, and PFKFB3 (Figures 3(a)–3(d)) expression when Kc7f2 was administered to BV2 cells. To further confirm that isoschaftoside can inhibit the upregulation of HIF-1*α*, BV2 cells were pretreated with CoCl_2_ for 24 h followed by coadministration with isoschaftoside for 9 h. CoCl_2_ causes accumulation of HIF-1*α* in microglia through inhibiting HIF-1*α* degradation [14]. As shown in Figures 4(a) and 4(b), the fluorescence intensity of HIF-1*α* increased significantly in CoCl_2_-treated cells, whereas isoschaftoside abolished this phenomenon. Consistent with the immunofluorescence assay, the protein levels of HIF-1*α*, HK2, and PFKFB3 in microglia were significantly increased after CoCl_2_ treatment, while these upregulations were mitigated by isoschaftoside (Figures 4(c)–4(f)).

### 3.4. Isoschaftoside Attenuates Lipopolysaccharide-Induced Phosphorylation of ERK1/2 and mTOR

**Figure 5:**
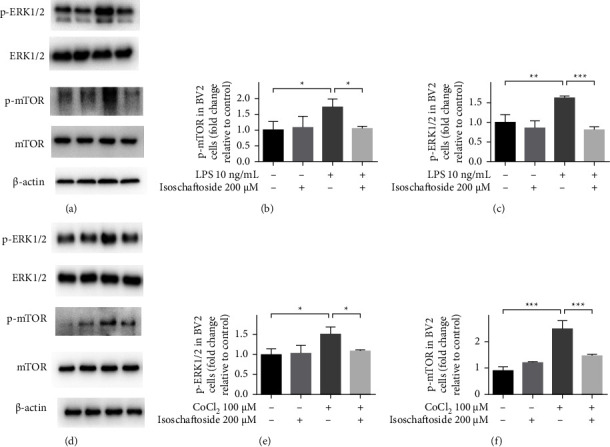
Isoschaftoside inhibits the activation of ERK1/2/mTOR pathway in microglia. (a–c) Expression of ERK1/2 and mTOR after 9 h of treatment with lipopolysaccharide (10 ng/mL) and isoschaftoside (200 *μ*M). (d–f) And when the cells were treated with CoCl_2_, isoschaftoside also inhibited the activation of ERK1/2/mTOR pathway in microglia. Data are means ± SD; ^*∗*^*P* < 0.05, ^*∗∗*^*P* < 0.01, ^*∗∗∗*^*P* < 0.001 for comparisons shown, *n* = 3. Statistical analyses were done by using two-way ANOVA with the Tukey's post hoc test.

Recent findings indicated that the ERK1/2/mTOR signaling pathway is involved in inflammatory response mediated by HIF-1*α* [15]. Therefore, the effects of isoschaftoside on protein expression and phosphorylation of ERK1/2 and mTOR pathways were analyzed. As shown in Figures 5(a)–5(c), lipopolysaccharide treatment resulted in significant increase in phosphorylation of ERK1/2 and mTOR, which were prevented by isoschaftoside administration (*P* < 0.05 or *P* < 0.001 versus CoCl_2_-treated cells). Similarly, phosphorylation of ERK1/2 and mTOR in microglia was significantly increased with CoCl_2_ (*P* < 0.05 or *P* < 0.001 versus control), and this upregulation was also mitigated by isoschaftoside (*P* < 0.05 or *P* < 0.001 versus CoCl_2_-treated cells, Figures 5(d)–5(f)).

## 4. Discussion

Activated microglia play an important role in the pathogenesis of neurodegenerative disorders such as Alzheimer's disease and Parkinson's disease [16, 17]. The current study demonstrated that isoschaftoside, a C-glycosyl flavonoid extracted from the root exudates of Desmodium uncinatum, prominently suppresses proinflammatory mediators' production in lipopolysaccharide-stimulated BV2 microglial cells.

**Figure 6:**
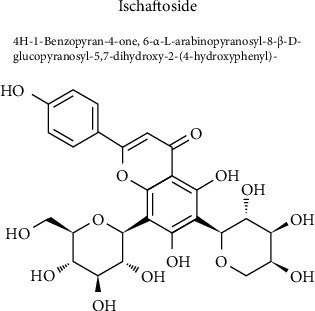
Chemical structure of isoschaftoside.

The regulatory effect of isoschaftoside on microglia activation may be related to its chemical structure (Figure 6). Flavonoids are reported to have a wide range of pharmacological activities, including blood lipid lowering, hypoglycemia, antiviral, neuroprotective, antioxidant, and antitumor activities [18]. Extensive evidence supports the anti-inflammatory features of numerous flavonoids in both preclinical and clinical studies [19]. For example, flavanone glycosides extracted from citrus fruits can reduce the levels of IL-1*β* and IL-6 in patients with neuritis [20]. In rat and mouse models of Parkinson's disease, the flavonoids have been shown to reduce astrogliosis, microgliosis, and the levels of proinflammatory mediators in the brain [21]. Flavonoid-rich ethanol extract from the leaves of Diospyros kaki can alleviate microglia and astrocyte activation, inhibit neuroinflammation, and attenuate neuronal apoptosis and synaptic dysfunction in D-galactose-aged mice [22]. Flavonoid-agathisflavone derived from the Brazilian plant *Poincianella pyramidalis* is able to regulate microglia polarization and promote remyelination [23]. To the best of our knowledge, this is the first report demonstrating that isoschaftoside is capable of regulating neuroinflammation in BV2 microglia.

The change of metabolic state recently has been suggested as an early inducement of neurodegenerative diseases [24]. The HIF pathway is one of the key transcriptional regulators in immunity and inflammation. Recent studies suggested that HIF-1*α* participates in the regulation of microglia metabolic reprogramming [24–26]. There are two key speed-limiting enzymes in the metabolic reprogramming pathway: PFKFB3 and HK2, which are closely related to the glycolysis transformation of lipopolysaccharide or hypoxia-activated microglia [27, 28]. We explored whether isoschaftoside inhibits lipopolysaccharide-induced microglia activation by inhibiting the expression of HIF-1*α*, the upstream regulator of glycolysis. Our results showed that the HIF-1*α* expression was significantly reduced in the isoschaftoside-treated microglia compared with lipopolysaccharide-stimulated cells. In addition, induction of HIF-1*α* accumulation by CoCl_2_ was also inhibited by isoschaftoside. Furthermore, the HIF-1*α* specific inhibitor Kc7f2 mitigated the metabolic reprogramming and anti-inflammatory effect of isoschaftoside. Thus, our results confirmed that HIF-1*α* is necessary for isoschaftoside to exert its anti-inflammatory effect.

ERK1/2 and mTOR have been suggested as important upstream pathways to induce HIF-1*α* transcription [29]. Therefore, it can be questioned whether the anti-inflammatory property of isoschaftoside is associated with regulation of ERK1/2 and mTOR in activated microglial cells. Our results showed that lipopolysaccharide-induced phosphorylation of ERK1/2 and mTOR in microglia was inhibited by isoschaftoside. A recent study suggested that schaftoside reduced neuroinflammation in BV2 microglia cells through the TLR4/MyD88-mediated mitochondrial fission pathway [30]. Therefore, it is possible that isoschaftoside regulates ERK1/2 and mTOR signaling pathways by binding to TLR4 in microglia as well.

The findings of this study have to be seen in light of possible limitation. The immortalized murine microglial cell line BV2 was used to construct an inflammatory model in our study. Although BV2 cells appear to be a valid substitute for primary microglia, however, the study indicated that BV2 cell responses to LPS and IL-4 were narrower and weaker than those of primary microglia [31]. Thus, the findings of this study need to be validated in primary microglia in the future.

In summary, the present study found that isoschaftoside prevents lipopolysaccharide-induced activation of ERK1/2/mTOR and HIF-1*α* mediated metabolic reprogramming, which leads to down-regulation of iNOS, COX-2, IL-1*β,* and TNF-*α* in BV2 microglial cells. Since isoschaftoside has very good bioavailability [32], our findings suggest that isoschaftoside may be a good medication for prevention or treatment of inflammation-related neurodegenerative diseases. Further preclinical and clinical studies are needed to better evaluate the potential therapeutic profile of isoschaftoside in neuroinflammation-mediated disorders.

## Data Availability

The data used to support the findings of this study are included within the article.
